# Combined X-ray and neutron single-crystal diffraction in diamond anvil cells

**DOI:** 10.1107/S1600576719014201

**Published:** 2020-02-01

**Authors:** Andrzej Grzechnik, Martin Meven, Carsten Paulmann, Karen Friese

**Affiliations:** a Institute of Crystallography, RWTH Aachen University, Jägerstrasse 17-19, Aachen, 52056, Germany; b Jülich Centre for Neutron Science (JCNS), Forschungszentrum Jülich GmbH at Heinz Maier-Leibnitz Zentrum (MLZ), Garching, 85748, Germany; c Mineralogisch-Petrographisches Institut, Universität Hamburg, Hamburg, 20146, Germany; d Jülich Centre for Neutron Science-2/Peter Grünberg Institut-4 (JCNS-2/PGI-4), Forschungszentrum Jülich GmbH, Jülich, 52425, Germany

**Keywords:** single-crystal X-ray diffraction, single-crystal neutron diffraction, high pressure, diamond anvil cells

## Abstract

It is shown that it is possible to perform combined X-ray and neutron single-crystal studies in the same diamond anvil cell (DAC). A modified Merrill–Bassett DAC equipped with an inflatable membrane filled with He gas has been developed. It can be used on laboratory X-ray and synchrotron diffractometers as well as on neutron instruments. The data processing procedures and a joint structural refinement of the high-pressure synchrotron and neutron single-crystal data are presented and discussed for the first time.

## Abstract 2

It is shown that it is possible to perform combined X-ray and neutron single-crystal studies in the same diamond anvil cell. The data processing procedures and a joint structural refinement of the high-pressure synchrotron and neutron single-crystal data are presented and discussed for the first time.

## 1. Introduction   

X-ray and neutron diffraction are complementary experimental techniques for detailed studies of crystalline materials. Neutron diffraction is indispensable when X-ray diffraction fails to probe, for instance, magnetic (dis)order (Devi *et al.*, 2019 ▸), compounds containing light elements (Truong *et al.*, 2018 ▸) or (dis)ordering of elements over crystallographic sites (Hering *et al.*, 2015 ▸). Because of the complementarity of X-ray and neutron data, their joint use allows the study of compounds in which magnetic order has a direct influence on the underlying crystal structure or *vice versa*, *e.g.* magnetocalorics (Hering *et al.*, 2015 ▸). In experimental electron-density studies of molecular crystals, the combination of X-ray and neutron single-crystal diffraction is frequent and often mandatory to fully understand the nature of bonding (Jarzembska *et al.*, 2017 ▸).

Owing to the development of radiation sources and area-sensitive detectors, single-crystal X-ray diffraction in a diamond anvil cell (DAC) can now be performed on very small samples (<10^−7^ mm^3^) with complex crystal structures up to megabar (1 Mbar = 100 GPa) pressures (Merlini & Hanfland, 2013 ▸). On the other hand, only two single-crystal neutron diffraction studies in a DAC have been reported, with complete structural refinements at 0.25 GPa (Binns *et al.*, 2016 ▸) and 1.0 GPa (Grzechnik *et al.*, 2018 ▸). The reason for this is that even at the most advanced neutron facilities it is difficult to study crystals with volumes below 1 mm^3^ since the highest neutron fluxes are several orders of magnitude smaller than the photon fluxes at synchrotron sources. Single crystals of several cubic millimetres are routinely studied with neutron scattering using gas-pressure and clamp cells (Klotz, 2013 ▸; Ridley & Kamenev, 2014 ▸). Data collected in Paris–Edinburgh presses are suitable for structure refinements at short neutron wavelengths (Bull *et al.*, 2011 ▸) but are very restricted in reciprocal space. Panoramic cells with large anvils made of sapphire (Kuhs *et al.*, 1989 ▸, 1996 ▸; McMahon *et al.*, 1990 ▸) or moissanite (McIntyre *et al.*, 2005 ▸) have been used to collect data for full structure refinements from neutron data. The future of high-pressure neutron scattering (including single-crystal diffraction) has recently been assessed by Guthrie (2015 ▸).

The requirement for large samples in neutron scattering hinders the joint use of X-ray and neutron single-crystal diffraction upon compression. Up to now, no complementary crystallographic studies at high pressures on exactly the same sample under exactly the same experimental conditions have been performed. In addition, DACs that are suitable for both X-ray and neutron diffraction studies are not commonly available.

Recently, we have started exploring the feasibility of neutron measurements in a DAC on the four-circle single-crystal diffractometer HEiDi (Meven & Sazonov, 2015 ▸) at the Heinz Maier-Leibnitz Zentrum (MLZ) in Garching. Using the hot source of the FRM II reactor, HEiDi operates at short monochromatic wavelengths with high fluxes. It is equipped with a point detector (Eurisys, 5 bar ^3^He) with a high sensitivity (>95%) down to λ = 0.3 Å. It provides precise information on crystal and magnetic structures, including the reliable and accurate characterization of anisotropic displacement parameters in materials with highly absorbing elements. Typical investigations at HEiDi focus on (i) ionic conductors relevant for energy applications and data storage (Ceretti *et al.*, 2018 ▸), (ii) superconductors (Jin *et al.*, 2016 ▸), (iii) multiferroic materials (Regnat *et al.*, 2018 ▸), (iv) small-mol­ecule structures, in which hydrogen bridges play a key role as a structure building element (Truong *et al.*, 2017 ▸, 2018 ▸), or (v) complex zeolitic crystal structures (Gatta *et al.*, 2018 ▸).

For our high-pressure work at HEiDi, we constructed a panoramic DAC with a wide access to reciprocal space (Grzechnik *et al.*, 2018 ▸). The data measured using this cell have a completeness of 76% (θ_full_ = 39.34°, λ = 1.17 Å) and are of excellent quality, as demonstrated by a full structure refinement with standard tools in high-pressure crystallography (Friese *et al.*, 2013 ▸; Petricek *et al.*, 2014 ▸). This cell is also compatible with the cryostats available at MLZ (Eich *et al.*, 2019 ▸). However, panoramic cells (regardless of whether the anvils are made of diamond, sapphire or moissanite), in which both incident and diffracted beams pass through the gasket, cannot be used for combined X-ray and neutron diffraction studies, because there is no gasket material that weakly attenuates neutrons and at the same time is transparent to high-energy X-ray radiation. Hence, a DAC working in the transmission mode is optimal for such experiments as the incident and diffracted beams pass through the diamond anvils, which (unlike sapphire and moissanite anvils) only weakly attenuate both X-rays and neutrons. The first cells for combined X-ray and neutron studies were developed by Goncharenko (2007 ▸). They are called hybrid DACs since they have windows for panoramic (neutron) and transmission (X-ray) geometries. However, these windows only allow measurement of the data at very small scattering angles owing to their limited opening angles. Consequently, comprehensive structural refinements are not possible either from the neutron or from the X-ray data obtained in the hybrid DAC because there are an insufficient number of accessible reflections.

Here, we present transmission cells suitable for both neutron and X-ray single-crystal diffraction with a comparatively large opening angle of 80°. They can be used on various diffractometers at laboratory X-ray, synchrotron and neutron facilities. The joint structural refinement of a crystal structure from the neutron and synchrotron data measured under identical conditions at high pressures is performed and discussed here for the first time.

For our combined benchmark X-ray and neutron measurements, we chose a crystal of MnFe_4_Si_3_ that has already been investigated in our earlier high-pressure neutron single-crystal diffraction study (Grzechnik *et al.*, 2018 ▸). The structure of this compound is well known and was determined at ambient conditions using joint X-ray and neutron data (Hering *et al.*, 2015 ▸). While the atomic coordinates can be straightforwardly obtained from X-ray diffraction, the occupation factors for the mixed occupancy site incorporating Mn and Fe (*i.e.* neighbouring elements in the periodic table) can only be determined from neutron diffraction since the two elements have very different scattering lengths.

## 2. The cells   

**Figure 1:**
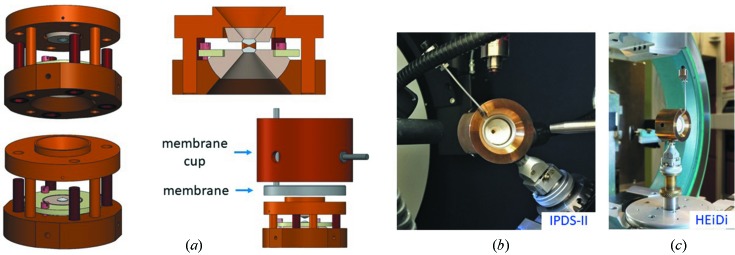
The cell used in this study: (*a*) a schematic cross section and different views with and without the membrane and cup (the valve is not shown), (*b*) the cell mounted on the laboratory X-ray diffractometer IPDS-II, and (*c*) the cell mounted on the four-circle diffractometer HEiDi.

The design of our transmission cells is a modification of the triangular DAC type designed by Merrill & Bassett (1974 ▸). They are round, with the central part having a fourfold symmetry, and fitted with conical diamonds (type I*a*, aperture 80°). The angular opening angle is 80°. The four guiding pins are integrated into the body of the cell. The smallest cell is 30 mm in diameter and 20 mm in height. Pressure is generated by tightening four M3 bolts. Like in the original Merrill–Bassett design (Merrill & Bassett, 1974 ▸), the diamond seats do not have any tilt adjustment. A larger cell, 44 mm in diameter and 25 mm in height [Fig. 1 ▸(*a*)], is equipped with a remotely inflatable membrane filled with He gas (memDAC). The diamonds can be aligned in both a parallel and a translational manner. Pressure in this cell is generated by tightening four M4 bolts and/or by inflating the membrane (LeToullec *et al.*, 1992 ▸; Goncharenko, 2007 ▸). A valve attached to the membrane through a capillary allows the cell to be disconnected from a pressure controller, while maintaining the pressure in the membrane. The size of the outside membrane cup is 50 mm in diameter and 33 mm in height. The outer dimensions of both cells and of the membrane cup are quite small so that the same crystal could be studied under the same conditions on laboratory X-ray and synchrotron diffractometers as well as on neutron beamlines [Figs. 1 ▸(*b*) and 1 ▸(*c*)]. Our memDAC is especially useful to follow the pressure dependence of selected reflections by using the remotely inflatable membrane at room temperature. The use of the membrane avoids safety concerns related to tightening the screws by hand when the sample might be activated by exposure to the neutron beam. The small size of the cells also makes them suitable for low-temperature investigations in closed-cycle cryostats when the membrane is not used.

All parts of the cells, except for the diamonds (https://diamondanvils.com/) and the membrane, capillary and valve (all three from https://www.betsa.fr/), are currently made of Berylco-25 (CuBe). The pressures reachable with diamond culets of 0.6 and 1.0 mm in diameter are above 10 and 4 GPa, respectively. Alternatively, the M3 and M4 bolts can be made of high-tensile titanium alloys (Klotz, 2013 ▸). The DAC can also be produced from Ni–Cr–Al alloy (‘Russian alloy’, NiCrAl), which has superior mechanical properties compared with CuBe (Cheng *et al.*, 2019 ▸). Unlike most of the materials that are used to manufacture DACs for X-ray diffraction, CuBe and NiCrAl are ideal for both X-ray and neutron diffraction as they are non-magnetic down to very low temperatures and weakly attenuate neutrons. Our cells made of CuBe do become activated by neutrons but always remain below the safety limits for free handling. In contrast, the most common maraging steels are not paramagnetic and become highly activated with neutrons as they have a significant content of cobalt.

## 3. Data collection and processing   

An approximately prismatic crystal of MnFe_4_Si_3_ (about 0.4 × 0.45 × 0.6 mm) was loaded into memDAC together with a ruby chip. The size of the crystal is only slightly larger than the minimal size for crystals that can currently be measured at HEiDi before all the upgrades of the beamline are finished (Grzechnik *et al.*, 2018 ▸). The diameter of the diamond culets was 1.5 mm. To load such a large crystal and avoid crushing it between the anvils, the gasket with an initial thickness of 1 mm was pre-indented to about 0.8 mm. The initial diameter of the sample chamber was 0.8 mm. With so thick a pre-indentation, the pressure is limited by the size of the crystal and stability of the gasket. We therefore performed our benchmark measurement at a relatively low pressure to ensure stable conditions for both neutron and X-ray measurements, despite the fact that the pressure limits for the cell are higher (see above).

To minimize the background due to neutron scattering from all the components of the cell, the conical surfaces of the seats and of both upper and lower parts of the cell were covered with a gadolinium paint, *i.e.* a fine powder of Gd_2_O_3_ mixed with nail polish [Figs. 1 ▸(*b*) and 1 ▸(*c*)]. As a result, the holes in the seats effectively acted as pinholes with a diameter of 3.2 mm, *i.e.* the diameter of the diamond table. The pressure determined using ruby luminescence was 0.9 GPa. The transmitting medium was a deuterated 4:1 mixture of methanol and ethanol.

Prior to the neutron measurements on HEiDi, we determined the orientation matrix of the crystal in the DAC on a Stoe IPDS-II laboratory single-crystal diffractometer (Mo *K*α) [Figs. 1 ▸(*b*) and 1 ▸(*c*)]. The collected data were processed with the software *X-Area* (Stoe & Cie, 2013 ▸).

Unlike in the original Merrill–Bassett type (Merrill & Bassett, 1974 ▸; Binns *et al.*, 2016 ▸), the use of the membrane precludes neutron data collection through the cell body of our memDAC. Hence, the diffracted neutron intensities (Table 1 ▸) were measured within the cones of the cell (the opening angle 80°). The HEiDi diffractometer has a four-circle Eulerian geometry. At the beginning of the neutron experiment (λ = 1.17 Å), memDAC was oriented with its axis coinciding with the primary beam, *i.e.* all diffractometer axes 2θ, ω, χ and φ were at their zero positions. The sample position in memDAC was optically adjusted to the instrument centre, defined by the cross point of all diffractometer axes.

Taking into account the relationship between the two reference systems of the IPDS-II and four-circle Eulerian diffractometers, we could deduce an approximate orientation matrix of the single crystal on HEiDi. This facilitated finding the reflections with the point detector. After subsequent centring of these reflections and refinement of the orientation matrix, we calculated the offset of the two diffractometers, which results in small deviations in the angular values of χ and φ in the Eulerian geometry of the four-circle diffractometer HEiDi with respect to the angular values deduced from the orientation matrix from the IPDS-II. Knowing the exact transformation between the two orientation matrices, we can now efficiently use the peak search routines in reciprocal space with the point detector on HEiDi by employing the orientation matrices obtained on our laboratory X-ray instrument in future experiments. In a similar way, it is possible to use the orientation matrices from other diffractometers, provided the transformation between the two reference systems is known. The small additional angular deviations can then be straightforwardly determined, leading altogether to a reduction of the measurement time compared with strategies where the classical search routines are used at HEiDi. These search routines can easily take up to 1 or 2 days of beamtime depending on the size of the crystal.

The software at HEiDi now includes a mathematical model describing the position of memDAC when rotating the sample. With the help of this algorithm, diffracted beams not passing through both diamonds in the cell but hitting the body are marked as ‘shaded’ and the corresponding reflections are excluded automatically from the measurement. The routine furthermore checks the possibilities to rotate the sample in the DAC around the **H**_*hkl*_ vector and determines whether an alternative azimuthal ψ angle is available where the shading can be avoided and the reflections are accessible.

For the primary neutron optics, a setup with a 0.5 mm Er filter to suppress λ/3 contamination and the adjustable 3 mm BN (boron nitride) pipe collimator was used. For the secondary optics, a 16 mm BC (boron carbide) tube for suppression of scattering from the sample environment and a detector slit of 10 × 15 mm (width × height) to minimize background were used. All reflections were measured with ω rocking scans of 10 s per step using a point detector. If significant (*I* > 3σ), they were re-measured for up to an additional 10 s per step in order to increase their accuracy. The combined effect of the diamond seat pinholes and ω scans was that the background due to powder lines originating from the sample environment was minimal. Because of the restrictions in beamtime and in the angular range of the DAC, only (±*h* +*k* ±*l*) reflections up to 2θ = 65° were considered for collection.

Synchrotron single-crystal experiments in memDAC (Table 1 ▸) were conducted on the beamline P24 (Chemical Crystallography) at PETRA-III (Hamburg) using a marCCD165 detector on the kappa diffractometer (station EH1, λ = 0.494 Å). The exposure time per frame was 1 s. A filter (an Ni foil with a thickness of 50 µm) was additionally used to attenuate the primary beam. The data were indexed and integrated with the software *CrysAlis* (Oxford Diffraction, 2007 ▸). The obtained lattice parameters and unit-cell volume are *a* = 6.7705 (6), *c* = 4.7044 (2) Å and *V* = 186.76 (4) Å^3^, respectively.

## 4. Structural refinements   

For all structural refinements with the program *JANA2006* (Friese *et al.*, 2013 ▸; Petříček *et al.*, 2014 ▸), the lattice parameters obtained from the synchrotron data were used because of their higher accuracy. The relatively high internal *R* values for both neutron and synchrotron data can possibly be attributed to the radiation attenuation by the diamonds, *i.e.* ‘diamond dips’ (Loveday *et al.*, 1990 ▸). Initially, the refinements of the synchrotron and neutron data were performed separately to test their internal consistency (Tables 1 ▸ ▸ ▸ ▸–5 ▸). Because of the limited number of reflections available (determined by the opening angle of the cell), the displacement parameters of the atoms were refined isotropically from the neutron data.

A comparison of the atomic coordinates from the present neutron data with those obtained previously (Grzechnik *et al.*, 2018 ▸) shows that they agree very well within their estimated standard deviations (ESDs) (Table 4 ▸). The interatomic distances are also identical within their ESDs to those from our panoramic cell (Table 5 ▸). On the other hand, the completeness of the data in memDAC is significantly lower. The fraction of unique reflections measured out to the angle θ_full_, *i.e.* to the angle at which the measured reflection count is close to being complete, is 62% at θ_full_ = 20.22° for memDAC compared with 76% at θ_full_ = 39.34° for the panoramic cell (Grzechnik *et al.*, 2018 ▸).

**Figure 2:**
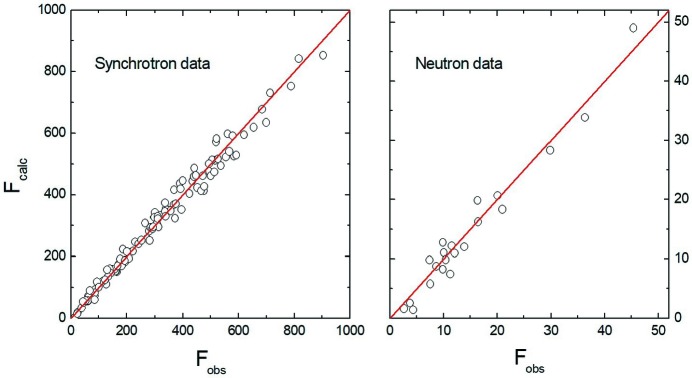
Observed (*F*_obs_) versus calculated (*F*_calc_) structure factors based on symmetry-independent reflections from a joint refinement of the synchrotron and neutron data.

In a second step, a joint refinement combining the synchrotron and neutron data using the available options in the program *JANA2006* was performed. The corresponding *F*_obs_ − *F*_calc_ plots are shown in Fig. 2 ▸. There are approximately ten times more reflections measured with the synchrotron radiation than with neutrons. As can be seen from Table 3 ▸, the atomic coordinates and displacement parameters in the joint refinement are identical to those from the refinement of the synchrotron data alone.

The refinement with only the synchrotron data showed that it was not possible to refine the Mn and Fe occupancies reliably, owing to the limited contrast of these neighbouring elements in X-ray diffraction. To distinguish these two elements, the information from the neutron data is clearly needed. Initially, Mn and Fe were equally distributed on the two available Wyckoff positions 6*g* and 4*d* (Table 4 ▸). The refinement of the occupancies yielded a model in which the position 6*g* is partially occupied by Mn and Fe, while the position 4*d* is essentially occupied by Fe. Despite the fact that only relatively limited neutron data are available, a comparison with earlier published structural data based on neutron single-crystal diffraction at ambient (Hering *et al.*, 2015 ▸) and high (Grzechnik *et al.*, 2018 ▸) pressures shows that taking them into account in the refinement still allows us to unambiguously refine the occupancies of Mn and Fe on the available sites in agreement with the earlier results (like in the earlier refinements, the sum of occupancies was restricted in such a way that the overall stoichiometry corresponded to the ideal one MnFe_4_Si_3_ in accordance with chemical analysis). It is noteworthy that a test carried out with the present neutron data shows that, even if only the ten strongest reflections are included, the occupancy factors are still correct and only their standard deviations become slightly higher. This implies that for answering other specific questions, regarding, for instance, magnetic ordering or the positions of hydrogen bonds, a comparatively small number of reflections measured with neutrons would also be sufficient if they are combined with X-ray data.

## 5. Conclusions   

Our study confirms that it is possible to determine the crystal structure of a compound under high pressure using a combination of neutron and X-ray data measured on the same crystal under identical conditions. The transmission DACs described in this study can be used on various diffractometers at laboratory X-ray, synchrotron and neutron facilities.

The major problem in high-pressure investigations using neutron single-crystal diffraction in DACs, especially using a point detector, is the large amount of time needed to measure a complete data set owing to the small sample volume and the comparatively low flux of the neutron beam. However, in many cases a full neutron data set does not necessarily have to be measured. Rather a small number of reflections, which are relevant to the scientific question one wants to answer, can be selected to reduce the required beamtime. Complementary data can then be measured with X-ray diffraction, for which the data collection is more efficient.

Currently, the opening angles of our transmission DAC are 80°, which is a standard in the DACs for single-crystal diffraction. We are now designing new cells made of the Ni–Cr–Al alloy (Cheng *et al.*, 2019 ▸) for neutron and X-ray diffraction with a larger opening that would allow a wider access to the reciprocal space, close to that in our panoramic DAC (Grzechnik *et al.*, 2018 ▸). The resulting improvement in the quality and redundancy of the neutron data will mitigate the effect of extinction and radiation attenuation by the diamond anvils. The latter has been treated semi-empirically for time-of-flight neutron diffraction (Guthrie *et al.*, 2017 ▸). It also remains to be accounted for in monochromatic neutron scattering.

## Supplementary Material

Crystal structure: contains datablock(s) global, neutron, global_2, synchrotron. DOI: 10.1107/S1600576719014201/pd5113sup1.cif

Structure factors: contains datablock(s) neutron. DOI: 10.1107/S1600576719014201/pd5113neutronsup2.hkl

Structure factors: contains datablock(s) synchrotron. DOI: 10.1107/S1600576719014201/pd5113synchrotronsup3.hkl

CCDC references: 1959957, 1959958

## Figures and Tables

**Table 1:** Experimental single-crystal data on MnFe_4_Si_3_ (*P*6_3_/*mcm*, *Z* = 2) measured with synchrotron and neutron diffraction

	Synchrotron	Neutron
Data collection		
No. measured reflections	509	56
Range of *hkl*	−10 ≤ *h* ≤ 9	0 ≤ *h* ≤ 6
	−7 ≤ *k* ≤ 4	−3 ≤ *k* ≤ 0
	−7 ≤ *l* ≤ 7	0 ≤ *l* ≤ 4
No. observed reflections†	106	15
*R*(int)_obs_ ‡	13.10	20.65
Redundancy	4.8	2.6

† Criterion for observed reflections is |*F*_obs_| > 3σ.

‡ All agreement factors are given in %, weighting scheme is 1/[σ^2^(*F*_obs_) + (0.01*F*_obs_)^2^].

**Table 2:** *R*-factor overview for the separate refinements of the synchrotron and neutron data

	Synchrotron	Neutron
*R*_obs_	6.21	7.00
*wR*_obs_	7.60	4.41
Goodness of fit	6.50	1.93
No. of parameters	12	7

**Table 3:** *R*-factor overview for the joint refinement of the synchrotron and neutron data (the number of refined parameters is 14)

	Block 1 (synchrotron)	Block 2 (neutron)
*R*_obs_	6.19	9.70
*wR*_obs_	7.58	7.10
Goodness of fit	6.02	7.74

**Table:** Italics: synchrotron diffraction (a separate refinement); normal font: neutron diffraction (a separate refinement); bold font: both synchrotron and neutron diffraction (a joint refinement).

Atom	Wyckoff position	Occupancy	*x*	*y*	*z*	*U*_eq_
Mn1	6*g*	*0.3333*	*0.7649 (3)*	*0.7649 (3)*	*0.25*	*0.0150 (8)*
		0.33 (2)	0.759 (3)	0.759 (3)	0.25	0.025 (9)
		**0.32 (2)**	**0.7649 (3)**	**0.7649 (3)**	**0.25**	**0.0151 (7)**

Fe1		*0.6667*	*0.7649 (3)*	*0.7649 (3)*	*0.25*	*0.0150 (8)*
		0.67 (2)	0.759 (3)	0.759 (3)	0.25	0.025 (9)
		**0.68 (2)**	**0.7649 (3)**	**0.7649 (3)**	**0.25**	**0.0151 (7)**

Fe2	4*d*	*1.0*	*0.6667*	*0.3333*	*0*	*0.0132 (6)*
		0.99	0.6667	0.3333	0	0.025 (8)
		**0.98**	**0.6667**	**0.3333**	**0**	**0.0132 (6)**

Mn2		*0.0*	*0.6667*	*0.3333*	*0*	*0.0132 (6)*
		0.01	0.6667	0.3333	0	0.025 (8)
		**0.02**	**0.6667**	**0.3333**	**0**	**0.0132 (6)**

Si	6*g*	*1.0*	*0.3998 (4)*	*0.3998 (4)*	*0.25*	*0.013 (1)*
		1.0	0.391 (6)	0.391 (6)	0.25	0.028 (8)
		**1.0**	**0.3998 (5)**	**0.3998 (5)**	**0.25**	**0.013 (1)**

**Table:** 

Atom	*U*_11_	*U*_22_	*U*_33_	*U*_12_	*U*_13_	*U*_23_
Mn1/Fe1	*0.016 (1)*	*0.010 (1)*	*0.017 (1)*	*0.0051 (6)*	*0*	*0*
	**0.016 (1)**	**0.10 (1)**	**0.017 (8) **	**0.0051 (5)**	**0**	**0**

Fe2	*0.013 (1)*	*0.013 (1)*	*0.013 (1)*	*0.0066 (6)*	*0*	*0*
	**0.013 (1)**	**0.013 (1)**	**0.013 (1)**	**0.0066 (5)**	**0**	**0**

Si	*0.012 (2)*	*0.010 (2)*	*0.017 (1)*	*0.0049 (9)*	*0*	*0*
	**0.012 (1)**	**0.010 (2)**	**0.017 (1)**	**0.0050 (8)**	**0**	**0**

**Table 5:** Selected interatomic distances (in Å) in MnFe_4_Si_3_

		Synchrotron	Neutron	Synchrotron and neutron
Mn1—Mn1	2×	2.757 (3)	2.83 (3)	2.757 (2)
Mn1—Mn1	4×	2.840 (1)	2.863 (9)	2.840 (1)

Mn1—Fe2	4×	2.902 (2)	2.88 (1)	2.902 (3)
Mn1—Si		2.472 (4)	2.49 (4)	2.472 (4)
Mn1—Si	2×	2.356 (3)	2.32 (3)	2.356 (3)
Mn1—Si	2×	2.603 (2)	2.56 (2)	2.603 (2)

Fe2—Fe2	2×	2.3522 (2)	2.3522 (2)	2.3522 (2)
Fe2—Si	6×	2.380 (3)	2.40 (4)	2.380 (3)

Si—Si	2×	2.715 (2)	2.77 (3)	2.715 (3)
